# The Pleiotropic Effect of Vitamin D

**DOI:** 10.5402/2013/898125

**Published:** 2013-09-04

**Authors:** Yu-Hsien Lai, Te-Chao Fang

**Affiliations:** ^1^Division of Nephrology, Department of Internal Medicine, Buddhist Tzu Chi General Hospital, No. 707, Section 3, Chung Yang Road, Hualien 97004, Taiwan; ^2^School of Medicine, Tzu Chi University, Hualien, Taiwan; ^3^Institute of Medical Sciences, Tzu Chi University, Hualien, Taiwan

## Abstract

The novel roles of vitamin D were discovered and valued in this century. In addition to the maintenance of calcium and phosphorus balance, vitamin D regulates the function of the kidneys, heart, and immune system. Moreover, its anti-inflammatory, antiapoptotic, and antifibrotic roles have gained considerable attention. Vitamin D is also important for the maintenance of homeostasis by regulation of hormone secretion, cell proliferation, and differentiation. This paper will review these pleiotropic functions of vitamin D.

## 1. Introduction

Since the beginning of the 20th century, scientists have been exploring the functions of vitamin D. The roles of this vitamin in endocrine system and metabolic bone diseases were already well studied by 1970. In this century, the discovery of vitamin D receptor has provided more insight on its additional functions [1]. Vitamin D receptors are present on many organs, such as the pancreas, large and small intestines, muscles, and nervous system [2]. Vitamin D was found to regulate the cell cycle and subsequently influence organ functions by binding to its receptor on the cells of the immune, nervous, and cardiovascular systems [3]. In the kidneys, vitamin D exerts protective effects by inhibiting renal fibrosis, inflammation, and progression of proteinuria.

Vitamin D deficiency is strongly associated with various cardiovascular and metabolic diseases such as hypertension, type 1 diabetes, myocardial infarction, and stroke. Moreover, vitamin D deficiency is related to several autoimmune diseases such as rheumatoid arthritis, systemic sclerosis, and systemic lupus erythematosus. Studies also have shown a negative correlation between serum vitamin D concentration and incidence of colorectal cancer and breast cancer [4]. These phenomena suggest that vitamin D plays protective roles in many diseases. As the importance of vitamin D for endocrine function has gained attention, the pursuit of paracrine and autocrine functions of vitamin D will continue in this century [5].

## 2. Metabolism of Vitamin D

Vitamin D is a fat-soluble vitamin produced by exposure of the skin to sufficient ultraviolet B radiation and absorption from the gastrointestinal tract. After vitamin D_3_ is synthesized, it is transported to the liver where 25-hydroxyvitamin D_3_ is formed via hydroxylation by 25-hydroxylase. 25-Hydroxyvitamin D_3_ is further converted into the physiologically active vitamin D_3_ (1,25-dihydroxyvitamin D_3_) in the mitochondria of the proximal convoluted tubules. The active vitamin D_3_ and vitamin D-binding protein are then transported to different organs for further metabolism [6]. In patients with chronic kidney disease, the serum level of the active form of vitamin D_3_ is decreased because of elevated blood concentration of fibroblast growth factor-23 (FGF-23) and related inflammatory cytokines [7, 8]. Because the level of circulating vitamin D_3_ decreases, the levels of 25-hydroxyvitamin D entering other types of cells also reduce relatively [9]. 

The daily recommended vitamin D intake is 5–15 *μ*g, but the amount of vitamin D from UV irradiation via skin or oral intake is inadequate to meet the demand for most people nowadays [5]. Because the incidence of vitamin D toxicity is rare in healthy adults, increased daily vitamin D intake is suggested [8, 10]. If the daily intake of vitamin D can reach 50 *μ*g, the concentration of serum vitamin D in the blood can increase from 25 nmol/L to 75 nmol/L [11]. Increasing vitamin D intake would be helpful in disease prevention and management [12]. 

## 3. Vitamin D Receptor 

The gene for vitamin D receptor was discovered in 1988 and has been found to be present in the cells of many tissues, including parathyroid cells, pancreatic cells, macrophages, keratinocytes, special nerve cells, and renal tubular cells. Vitamin D receptor is widely expressed in almost all cells, and vitamin D regulates approximately 3% of the human genes via its endocrine effects [13]. The active form of vitamin D is released in smooth muscle, colon, and immune cells, besides renal cells, via local hydroxylation of 25-hydroxyvitamin D by 1*α*-hydroxylases [14, 15]. 

## 4. Anti-Inflammatory Effect of Vitamin D 

One of the functions of vitamin D is to promote the differentiation of monocytes into macrophages, dendritic cells, and lymphocytes. These cells represent the first line of defense of the nonspecific immune system and play an important role in infection control [10]. Many studies have found that the lack of vitamin D or vitamin D receptor causes altered innate and adaptive immune functions. Patients with diseases associated with vitamin D deficiency, such as rickets or chronic kidney disease, are known to have recurrent infections [16, 17]. The effect of vitamin D on immune system can be attributed to the paracrine feedback mechanism, whereby it reduces inflammatory response, affects the differentiation of active CD4^+^ T cells, and enhances the inhibitory function of T cells. The active form of vitamin D also promotes the differentiation of monocytes into mature macrophages by induction of p21 [18]. C/EBP*β* (CCAAT-enhancer-binding protein beta) is an important transcriptional factor which provides macrophages with antibacterial, antiviral, and antitumor activities and for the IL-12 synthesis [19]. Vitamin D induces C/EBP*β* that contributes to the monocyte-macrophage lineage differentiation, increases the activity of macrophages, and promotes their cytotoxicity. Therefore, vitamin D enhances host defense against bacterial infections, as well as growth of tumor cells [20].

In 2007, Schauber et al. found that vitamin D can stimulate human skin cells to synthesize the antimicrobial peptide cathelicidin, which can enhance the innate immune function [21]. The active vitamin D-vitamin D receptor complex was found to influence *Mycobacterium tuberculosis* infection mainly by inhibiting the synthesis of IL-12 and *γ*-interferon, as well as the Th1 immune responses [22]. A meta-analysis study showed that the serum 25-hydroxyvitamin D concentrations were significantly lower in patients with tuberculosis than in the control group [23]. Vitamin D deficiency has also been found to be associated with increased incidence of respiratory diseases, such as influenza; *Mycobacterium tuberculosis* infection; and chronic respiratory diseases, such as cystic fibrosis, interstitial lung disease, and chronic obstructive pulmonary disease. 

The active vitamin D has also been found to have inhibitory effects on transplant rejection. Studies on heart transplantation have shown that active vitamin D may be more effective than cyclosporine in prolonging the survival of the transplanted organ and will not increase the rate of infection [24]. In kidney transplantation, the active vitamin D also extends the viability of the transplanted kidney and reduces the progression of renal fibrosis [25]. The above antirejection effect occurs through the TGF-*β*/Smad3 pathway [26].

## 5. Antiapoptotic and Antifibrotic Effects of Vitamin D

In normal tissues, vitamin D plays an important role in regulating the proliferation by promoting apoptosis. For example, in breast tissue, vitamin D regulates apoptosis according to the requirements of the body at different physiological stages such as pregnancy and breastfeeding [27]. In addition to the normal tissues, vitamin D has been reported to be important in the regulation of hyperplasia in cancerous and noncancerous tissues via initiation of apoptosis in glioma, melanoma, and breast cancer cells [28]. In breast cancer cells, vitamin D induced apoptosis via interaction between Bcl2 and Bax [29]. In colorectal cancer, the transcription factor Snail reduces the expression of the vitamin D receptor, thereby influencing the progression of colon cancer cells [30]. The amount of vitamin D receptor is an important factor in determining its potency in the regulation of tumor growth.

In the nervous system, the active vitamin D affects the conduction of the motor neurons and synthesis of neurotrophic factors, thus preventing damages to the neurons [31].

Further, excess formation of keratin in psoriasis is due to the overexpression of TGF-*α*. Vitamin D helps in reducing the proliferation of keratinocytes, hence treating psoriasis by inhibiting the growth cycle of the TGF-*α*/EGFR (epidermal growth factor receptor) [32].

## 6. Vitamin D in Kidney Disease, Diabetes Mellitus, and Cardiovascular Disease

Active vitamin D has a negative feedback on the renin-angiotensin system, which plays a key role in regulating blood pressure, electrolyte levels, and volume status. When patients have low serum levels of active vitamin D, they may develop high blood pressure or diseases related to high plasma renin activity [33]. Studies on knockout mice lacking active vitamin D receptor expression revealed elevated levels of renin and angiotensin II in the blood, which in turn caused a significant increase in blood pressure, cardiac hypertrophy, and water retention [34].

Calcitriol, an analogue of the active vitamin D, exerted inhibitory effects on renal interstitial myofibroblasts and thereby inhibited the progression to renal interstitial fibrosis [35]. Several studies on nephropathy showed that active vitamin D protects the kidneys through its anti-inflammatory and antifibrotic effects [36, 37]. Vitamin D deficiency has also been found to be associated with earlier-onset and highly severe diabetes mellitus, presumably because of abnormal insulin secretion and immune dysfunctions. The condition of such diabetes patients can be improved by calcitriol supplementation [38]. A UK population-based study found that patients with type 1 diabetes had lower serum 25-hydroxyvitamin D concentrations than did healthy subjects of the same age. The study also found that the 3 main genes controlling the 25-hydroxyvitamin D metabolism are related to the incidence of type 1 diabetes [39]. Both *in vitro* and *in vivo* studies also showed that vitamin D could prevent the destruction of pancreatic beta-cells and reduce the incidence of autoimmune diabetes mellitus, possibly secondary to inhibition of proinflammatory cytokines, such as tumor necrosis factor (TNF-*α*) [5].

In the cardiac system, vitamin D maintains cardiovascular health by direct binding to vitamin D receptor on the myocardial cells, and thus regulating the hypertrophy of myocardial cells and the synthesis and release of atrial natriuretic peptide [40, 41]. Vitamin D has been shown to inhibit angiogenesis and increase matrix G1A protein synthesis and thus inhibit the synthesis of inflammatory cytokines such as tumor necrosis factor and interleukin [42]. On the other hand, vitamin D inhibits the calcification of blood vessels by regulating the activities of interleukins [43]. In patients with end-stage renal disease, vitamin D supplementation has been found to improve left ventricular function and muscle weakness, but the mechanism underlying this function is not known yet [11]. Vitamin D deficiency has been found to be associated with a variety of cardiovascular and other diseases, such as hypertension, diabetes mellitus, myocardial infarction, stroke, congestive heart failure, peripheral vascular disease, and atherosclerosis [12]. Therefore, the serum level of vitamin D is considered to be an important independent predictor of cardiovascular diseases [5].

## 7. Vitamin D in the Immune System

The interaction of vitamin D with the immune system is one of its most well-known effects [44]. The active vitamin D regulates innate and adaptive immune system, because its receptors are widely present on many immune cells, such as macrophages, dendritic cells, T cells, and B cells [45]. Vitamin D is thought to be able to activate cathelicidins, antimicrobial peptides present within the lysosomes of macrophages, and polymorphonuclear leukocytes [46]. Cathelicidins play a key role in innate immune defense against bacterial infections [47]. Cathelicidins regulate the transcription of vitamin D receptor as its gene promoter contains the functional response to vitamin D [48]. The active vitamin D regulates this antimicrobial peptide function in many different types of cells, including macrophages, keratinocytes, lung epithelial cells, placental trophoblast cells, and myeloid cell lines [21, 49, 50]. Therefore, active vitamin D has been found to inhibit the initiation of many diseases, such as experimental autoimmune encephalomyelitis, thyroiditis, type 1 diabetes mellitus, inflammatory bowel disease, systemic lupus erythematosus, and Lyme arthritis [16, 51].

*In vitro* studies on systemic lupus erythematosus revealed that the abnormal immune response may be reversed by addition of vitamin D; therefore, vitamin D deficiency is considered to be associated with loss of immune tolerance [52]. Studies on rheumatoid arthritis found that the disease activity is negatively correlated with serum vitamin D concentration, and such a correlation is independent of the parathyroid function [53].

## 8. Vitamin D and Cancer

Several studies have shown that vitamin D plays a protective role in several types of cancer, such as prostate, breast, and colon cancer [10]. Vitamin D has also been found to inhibit proliferation of a variety of human leukemia cell lines and induce differentiation of normal and leukemic myeloid precursor, thereby increasing maturation and decreasing aggressiveness of potential leukemic cells. Therefore, vitamin D is helpful in the treatment of leukemia and other myeloproliferative disorders [54].

The state of knowledge on the protective effects in cancer of vitamin D is as follows.

1. Active vitamin D promotes the transcription of the cyclin-dependent kinase inhibitor p21 [18]. This is sufficient to suppress growth of cells of the monocyte-macrophage lineage and promote their differentiation.
2. Active vitamin D induces the synthesis of the cyclin-dependent kinase inhibitor p27 [55].
3. The proliferation of tumor cells is due to the overexpression of the TGF-*α*/EGFR pathway. Active vitamin D could inhibit the TGF-*α*/EGFR growth pathway [32].
4. In human epithelial cell tumors, C/EBP*β* is considered to be effective in the inhibition of the carcinogenic cell cycle protein D1 [56]. In contrast, the C/EBP*β* isoform LIP can enhance the activity of the carcinogenic cyclin D1 and induce cell growth. Therefore, the proliferative property of human tumors is inversely correlated to the intracellular C/EBP*β*-to-LIP ratio [57]. The active vitamin D can induce the expression of C/EBP*β* and prevent the proliferation of LIP epidermal growth factor receptor, thus reducing the occurrence of EGFR-driven related cancers [58].
5. Vitamin D plays a major role in cell metabolism as it regulates cell maturation, differentiation, and apoptosis [10]. These features are related to the suppressed expression of antiapoptotic proteins such as Bcl2 in cancer cells and arrest of cell cycle in G0/G1, which reduces the rate of proliferation [59]. Vitamin D was also found to have anti-inflammatory effects that can delay and prevent the development of cancers [60].

Studies have found that the adequacy of the content of vitamin D in the body is an important factor in predicting several types of cancer prognosis and mortality [5].

## 9. Conclusions

In the past decades, the function of vitamin D has been more deeply understood. The discovery of the vitamin D receptor enabled further investigations on the association of acute and chronic diseases with vitamin D deficiency. The pleiotropic effects of vitamin D and associated mechanisms are summarized in Table 1. In addition, the paracrine and autocrine effects of vitamin D have a protective role in many diseases. Therefore, the application of vitamin D in disease treatment and prevention should be pursued.

## Figures and Tables

**Table 1:** The pleiotropic effects of vitamin D and associated mechanisms and diseases.

Pleiotropic effects	Mechanism	Associated diseases
Anti-inflammation	(1) affects the differentiation of active CD4^+^ T-cells (2) enhances the inhibitory function of T-cells (3) promotes differentiation of monocyte into mature macrophages by inducing p21 (4) induces C/EBP*β* which contribute to the monocyte-macrophage lineage differentiation, increase the activity of macrophages, and promote their antibacterial and antiviral activities (5) inhibits the synthesis of IL-12, *γ*-interferon, and Th1 immune responses (6) inhibits TGF-*β*/Smad3 pathway on transplant rejection	(1) recurrent infections in rickets or CKD patients (2) increased incidence of respiratory diseases, such as influenza, mycobacterium tuberculosis, and chronic respiratory diseases, such as cystic fibrosis, interstitial lung disease, and chronic obstructive pulmonary disease

Antiapoptosis and antfibrosis	(1) induce apoptosis via interaction between Bcl2 and Bax in breast cancer cells (2) affect the conduction of the motor neurons and synthesis of neurotrophic factors, thus preventing damage of the neurons (3) inhibit the growth cycle of the TGF-*α*/EGFR and reduce the proliferation of keratinocytes	(1) progression of cancer cells (2) excess formation of keratin in psoriasis

Cardiovascular diseases	(1) have negative feedback on renin-angiotensin system in regulating blood pressure, electrolyte and volume status (2) have direct binding to vitamin D receptor on the myocardial cells and regulate the hypertrophy of myocardial cells (3) have synthesis and release of atrial natriuretic peptide (4) inhibit angiogenesis and increase matrix G1A protein synthesis, thus inhibiting inflammatory cytokines such as tumor necrosis factor and interleukin (5) inhibit calcification of blood vessels by regulating interleukins	(1) hypertension, water retention (2) cardiac hypertrophy, myocardial infarction, stroke, congestive heart failure, peripheral vascular disease, and atherosclerosis

Kidney diseases	(1) inhibit renal interstitial myofibroblasts, inhibiting the progression to renal interstitial fibrosis	(1) renal fibrosis

Diabetes mellitus (DM)	(1) prevents the destruction of pancreatic beta-cells (2) reduces autoimmune diabetes mellitus, possibly secondary to inhibition of proinflammatory cytokines, such as tumor necrosis factor (TNF-*α*)	(1) earlier onset and more severe DM (2) type I DM

Immune system	(1) activates cathelicidins, an antimicrobial peptide within the lysosomes of macrophages and polymorphonuclear leukocytes	(1) increases initiation of experimental autoimmune encephalomyelitis, thyroiditis, type 1 diabetes mellitus, inflammatory bowel disease, systemic lupus erythematosus, and Lyme arthritis (2) increases the disease activity of rheumatoid arthritis

Cancers	(1) promote the transcription of cyclin-dependent kinase inhibitors, p21 (2) induce synthesis of the cyclin-dependent kinase inhibitors, p27 (3) inhibit the TGF-*α*/EGFR growth pathway (4) induce the expression of C/EBP*β* and prevent proliferation of LIP epidermal growth factor receptor, thus reducing EGFR-driven related cancers (5) suppress expression of anti-apoptotic proteins such as Bcl2 of cancer cells, arrest of cell cycle in G0/G1, thus slowing proliferation of cancer cells	(1) prostate, breast, and colon cancers (2) leukemia and other myeloproliferative disorders
